# A 3.2 Gb/s SPAD-Based Multi-Channel QRNG Chip with Low-Optical-Power Operation via Rolling-Sampling Temporal Decorrelation

**DOI:** 10.3390/s26196350

**Published:** 2026-10-08

**Authors:** Tuo Zhou, Jiachang Li, Chao Luo, Zirui Zhang, Xiangshun Kong, Cheng Mao, Feng Yan

**Affiliations:** School of Electronic Science and Engineering, Nanjing University, Nanjing 210023, China; 522025230187@smail.nju.edu.cn (T.Z.); li.jia.chang@smail.nju.edu.cn (J.L.); lc3304344175@163.com (C.L.); ziruizhang@smail.nju.edu.cn (Z.Z.);

**Keywords:** quantum random number generator (QRNG), single-photon avalanche diode (SPAD), hardware security

## Abstract

Random numbers play a vital role in modern information security, where quantum random number generators (QRNGs) based on single-photon avalanche diodes (SPADs) offer a promising physical platform for quantum random-number generation. However, practical SPAD QRNGs can exhibit temporal correlation arising from finite photon-event statistics and detector nonidealities. To overcome these limitations, this paper presents a high-speed, 256-channel SPAD QRNG chip, featuring a 256 × 512-pixel array integrated with a rolling-sampling time-division multiplexing architecture. By virtue of the rolling-sampling mechanism, the single-pixel sampling interval is extended to 40.96 μs, which substantially reduces the measured temporal correlation by increasing the per-pixel revisit interval while preserving aggregate throughput. Within one complete operating cycle, the chip generates 256 random bits simultaneously, achieving a maximum aggregate raw output bitrate of 3.2 Gb/s without digital post-processing. An FPGA-based test platform was constructed for experimental verification. Measurements demonstrate that under standard indoor illumination (8 W/m^2^), the generated raw bitstreams pass all 15 NIST SP 800–22 statistical randomness tests at 3.2 Gb/s without post-processing. Furthermore, under controlled 530 nm illumination, the measured lag-1 serial autocorrelation coefficient decreases below the 10^−3^ reference level at an incident optical power of 11.6 μW. The proposed chip offers a low-optical-power, high-throughput solution for integrated hardware security.

## 1. Introduction

In today’s highly information-driven world, cryptography is inextricably linked to our daily lives, where random numbers serve as a fundamental security primitive for unpredictability [1]. Traditional true random number generators based on classical physical noise sources often suffer from environmental sensitivity and require complex post-processing to eliminate bias and correlation [2], which severely restricts the overall output bit rate.

Quantum random number generators, particularly those based on single-photon avalanche diodes (SPADs), have emerged as a highly attractive alternative due to their high scalability and stability [3]. Leveraging the independence of photons in a Poisson distribution within the light source, QRNGs can generate uncorrelated true random bits using SPADs in conjunction with photon counters [4] or timestamp circuits [5]. Relevant studies have modeled and analyzed these systems, achieving a series of advancements [6,7].

Despite the advantages, practical SPAD-based QRNG implementations can exhibit temporal correlation arising from finite photon-event statistics and detector nonidealities. Temporal correlation can arise from insufficient photon-event accumulation between successive samples, while detector nonidealities such as SPAD dead time further modify the registered-event statistics [8]. In conventional QRNG architectures operating with narrow sampling intervals, this dead-time memory effect induces strong serial autocorrelation. To reduce temporal correlation without sacrificing output rate, prior implementations have employed high photon-detection rates using externally driven optical sources [9]. Such illumination requirements may increase the complexity of the optical source and system integration.

To investigate low-optical-power operation while maintaining multi-gigabit throughput, this paper presents a high-speed, 256-channel SPAD QRNG chip fabricated in a standard CMOS process. The chip features a 256 × 512-pixel array integrated with a rolling-sampling time-division multiplexing readout architecture across 512 columns. By virtue of the rolling-sampling operation, the sampling interval for any individual pixel is extended to 40.96 μs, which is over two orders of magnitude larger than the SPAD dead time (∼30 ns). This spatial–temporal multiplexing increases the per-pixel revisit interval while preserving parallel output operation, thereby substantially reducing temporal correlation in the sampled raw sequence. Consequently, the chip achieves an aggregate raw output bitrate of 3.2 Gb/s without digital post-processing under indoor illumination while demonstrating low-optical-power operation at a tested incident power of 11.6 μW under 530 nm illumination.

The remainder of this paper is organized as follows. Section 2 formulates the fundamental SPAD-QRFF model and derives the rolling-sampling temporal decorrelation theory. Section 3 details the high-efficiency SPAD device structure, pixel logic, and the 256-channel rolling-sampling readout architecture. Section 4 describes the overall system design, board-level implementation, and experimental testing platform. Section 5 presents the chip testing, theoretical model validation, NIST randomness verification, and performance evaluation with varying light intensities. Finally, Section 6 concludes the paper.

## 2. SPAD-QRFF and Rolling-Sampling Temporal Decorrelation Model

### 2.1. SPAD-QRFF Model

**Figure 1:**
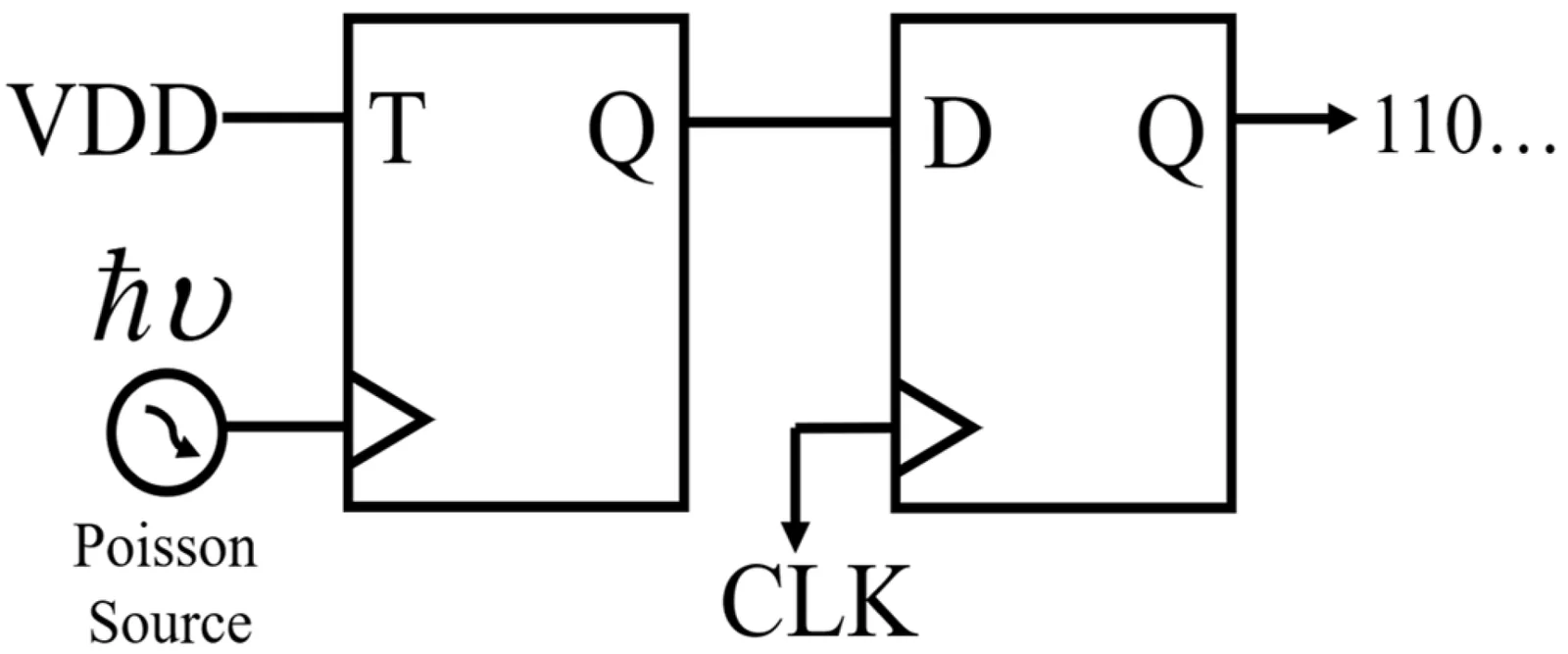
Random number generator model diagram, composed of a T flip-flop and a D flip-flop.

The quantum random flip-flop (QRFF) architecture leverages the intrinsic quantum randomness of single-photon avalanche diodes (SPADs) to generate random sequences [6]. As illustrated in Figure 1, the core mechanism comprises a trigger toggle flip-flop (TFF) and a sampling D-type flip-flop (DFF).

Photon arrivals at the SPAD are governed by a temporal Poisson process with an arrival rate of λ [10]. Each SPAD avalanche event generates a narrow active pulse that serves as the clock signal for the TFF. Driven by this Poisson arrival process, the TFF output transitions randomly over time, realizing a continuous-time random telegraph signal (RTS), denoted by S(t) ∈ {0,1} [11]. For an ideal Poisson process with rate λ, the probability of registering k avalanche events within a time interval τ is given by

(1) $$\;P(k)=\frac{{\left(\lambda \tau \right)}^{k}e^{-\lambda \tau }}{k!}$$

The RTS state at t + τ remains identical to its state at t if and only if an even number of TFF state transitions occur in [t,t + τ). Conversely, the state toggles if the number of transitions is odd. The ideal continuous-time autocorrelation function R_xx_(τ) of the RTS is derived as the expectation of state alignment:

(2) $$\;R_{xx}(\tau )={\sum_{k=0}^{\infty }\left(-1\right)}^{k}\frac{{\left(\lambda \tau \right)}^{k}e^{-\lambda \tau }}{k!}=e^{-2\lambda \left|\tau \right|}$$

To obtain a discrete binary sequence, the DFF latches the instantaneous state of S(t) at the rising edges of a periodic sampling clock with a period of T_SAMP_ [12]. The theoretical 1-bit lag serial correlation coefficient a1 of the sampled sequence is

(3) $$\;a_{1}=R_{xx}(T_{SAMP})=e^{-2\lambda T_{SAMP}}$$

While Equations (1)–(3) describe the ideal statistical behavior, physical circuit implementations introduce non-idealities that degrade sequence entropy. At the circuit level, real flip-flops exhibit finite and asymmetric rise and fall transition times (tr and tf). Defining η as the normalized sampling threshold voltage of the DFF, the statistical bit bias b (defined as b = P(X = 1) − 0.5) resulting from this edge mismatch is expressed as

(4) $$\;b=\frac{t_{f}-\eta (t_{r}+t_{f})}{2}\cdot \lambda$$

Equation (4) mathematically demonstrates that any mismatch between the rise and fall transition times (t_r_ ≠ t_f_) directly generates a proportional bit bias b that scales linearly with the photon detection rate λ. Minimizing the rise–fall time difference at the transistor level is therefore essential to eliminate statistical bias before sampling.

Beyond circuit-level edge mismatches affecting bias, detector-level non-idealities, primarily the SPAD dead time τ_d_, disrupt the pure Poisson arrival assumption of Equation (1). This detector memory modifies the registered-event statistics relative to the ideal Poisson model, such that Equation (3) no longer provides a complete description of the practical detector.

### 2.2. Rolling-Sampling Temporal Decorrelation Mechanism and Statistical Modeling

To incorporate the non-ideal counting behavior of the practical SPAD, the avalanche detection process for a non-paralyzable dead time τ_d_ is modeled as a stationary renewal process. For this model, the interval between two consecutive registered avalanche events can be expressed as

(5) $$\;X_{i}=\tau_{d}+E_{i}$$

where E_i_ is an exponentially distributed random variable with parameter λ_A_, representing the waiting time for the next photon arrival after the detector becomes active. The corresponding effective registered detection rate is therefore

(6) $$\;\lambda_{D}=\frac{\lambda_{A}}{1+\lambda_{A}\tau_{d}}$$

Unlike the ideal Poisson process considered in Section 2.1, the finite dead time introduces a temporal dependence between successive registered events. Accordingly, the parity correlation of the TFF output should generally be described from the counting statistics of the renewal process rather than from the ideal Poisson expression in Equation (3). For a stationary renewal process, the parity-dependent correlation can be written in the general form

(7) $$\;R_{XX}(\tau )=E[{\left(-1\right)}^{N(t,t+\tau )}]=\sum_{k=0}^{N}{\left(-1\right)}^{k}P_{k}(\tau )$$

where N(t,t + τ) denotes the number of registered avalanche events in the interval [t,t + τ), and P_k_(τ) is the probability of observing exactly k registered events within this interval. Equation (7) does not assume Poisson counting statistics and therefore provides a general description of the parity response for the renewal process.

In the following analysis, the detection process is assumed to be in its stationary regime after the initial transient has decayed. In particular, the observation is not initialized immediately after a specific avalanche event; instead, the initial residual waiting time is assumed to follow the equilibrium residual-life distribution of the stationary renewal process. This assumption avoids introducing an artificial correlation associated with a specially initialized renewal interval.

The proposed rolling-sampling architecture increases the temporal separation between two consecutive observations of the same SPAD pixel. For an array with C columns and a column scanning period T_COL_, the effective sampling interval of each pixel is

(8) $$\;T_{SAMP}=C*T_{COL}$$

Therefore, increasing the number of columns simultaneously changes two aspects of the sampled counting process. First, the sampling interval becomes much longer than the intrinsic detector dead time, thereby reducing the relative influence of dead-time-induced temporal dependence on the sampled sequence. Second, a longer sampling interval accumulates a larger number of photon-detection events, which changes the even-odd statistics of the accumulated count. These two effects are coupled through T_SAMP_ and are not independently quantified in the present work.

It should be emphasized that the renewal-process counting statistics do not, in general, satisfy the Poisson condition Var(N) = E[N], even when the observation interval is sufficiently long. Likewise, the asymptotic Gaussian behavior of long-window renewal counts does not imply Poisson counting statistics or directly determine the parity correlation of the TFF output. Therefore, the Gaussian asymptotic approximation is not used here to derive the parity-correlation expression.

For a tractable first-order description of the experimentally observed correlation-decay trend, the count accumulated within a sufficiently long sampling interval is instead approximated by an effective-Poisson random variable with mean λ_D_T_SAMP_. With this approximation, N_SAMP_ ∼Poisson(λ_D_ T_SAMP_). The parity characteristic function of a Poisson random variable is

(9) $$\;E[{\left(-1\right)}^{N_{SAMP}}]=\sum_{k=0}^{\infty }{\left(-1\right)}^{k}\frac{{\left(\lambda_{D}T_{SAMP}\right)}^{k}}{k!}e^{-\lambda_{D}T_{SAMP}}=e^{-2\lambda_{D}T_{SAMP}}$$

Thus, the one-bit-lag serial correlation can be approximated by

(10) $$\;a_{1}^{EP}\approx e^{-2\lambda_{D}T_{SAMP}}=e^{-2\lambda_{D}\cdot C\cdot T_{COL}}$$

where the superscript “EP” denotes the effective-Poisson approximation.

Equation (10) should therefore be interpreted as a first-order analytical reference rather than as an exact consequence of the renewal-process model. In particular, it is derived from the additional effective-Poisson assumption applied to the accumulated count and is not obtained by converting the Gaussian asymptotic statistics of the renewal process into Poisson statistics. Its applicability is expected to improve when the sampling interval is sufficiently longer than the characteristic detector-memory timescale and when the accumulated number of detection events within each sampling window is sufficiently large. Conversely, at short sampling intervals, residual detector and circuit memory can cause measurable deviations from the effective-Poisson relation.

In a practical SPAD implementation, such deviations may arise from non-ideal mechanisms that are not explicitly included in the first-order model, including detector afterpulsing associated with carrier trapping and release, quenching and reset dynamics, circuit-level memory, and other implementation-dependent effects. Therefore, Equation (10) is intended to capture the overall correlation-decay trend with increasing T_SAMP_ rather than to provide an exact prediction of the measured correlation at every sampling interval.

The proposed rolling-sampling scheme consequently provides a hardware-level means of increasing T_SAMP_ while maintaining parallel output operation. As T_SAMP_ increases, the combined effects of reduced relative dead-time influence and increased photon-count accumulation substantially suppress the temporal autocorrelation of the generated raw bits, thereby reducing the dependence on subsequent digital post-processing.

## 3. Chip Design

Building upon our previous research, we designed a random number generation chip for SPAD based on SMIC process technology. This chip features a pixel array size of 256 × 512, with each pixel serving as the smallest unit for generating random bits. The chip’s I/O circuitry controls the output, aggregating the random bits generated by each pixel into a continuous random bit sequence. The internal pixel architecture and structure of this chip are detailed in the subsequent sections.

### 3.1. Pixel Design

**Figure 2:**
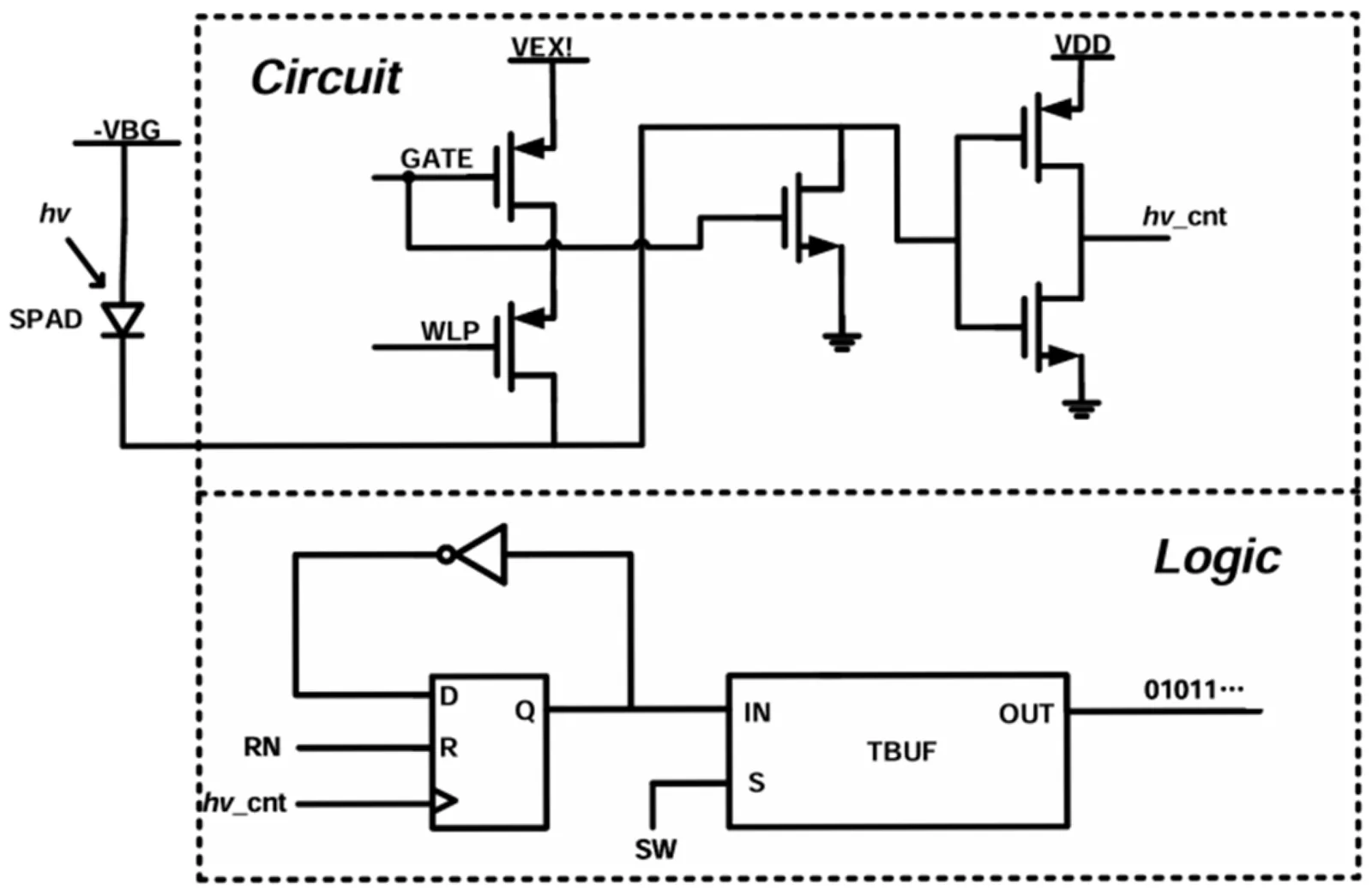
Pixel structure diagram. The entire pixel comprises two sections: the upper section contains the quenching circuit and waveform integration circuit, while the lower section houses the logic circuit.

The internal circuit structure of the pixel is shown in Figure 2. The upper half comprises the quenching circuit for the SPAD device, featuring five voltage inputs: VDD, VEX!, WLP, GATE, and VBG. VDD represents the power supply voltage required by the circuit, which is process-dependent. VEX! is the operating voltage for the signal processing circuit, typically around 2.5 V. WLP connects to the gate of a MOSFET, which operates in the linear region to provide quenching resistance; thus, WLP adjusts the resistance value of this MOSFET [13,14]. The VBG signal is a negative voltage that provides reverse bias to the SPAD device. The GATE voltage is the gate switching signal. When the chip is working, the GATE signal is low, enabling the SPAD device to operate normally in passive quenching mode [15], allowing photon capture. The design of the quenching circuit draws upon our previous research [16]. Following the quenching circuit is a D flip-flop connected to TBUF. This D flip-flop incorporates a reset switch controlled by RN. Its output passes through an inverter before connecting to the input terminal, while the quenching circuit’s output connects to the clock input of the D flip-flop. The D flip-flop’s output connects to TBUF’s input. TBUF incorporates a SW signal as its control input. TBUF’s output generates random bits. After sampling the SW signal, it resamples after an interval, continuously outputting random bits.

This pixel structure in Figure 2 is simple and similar to some published structures [17]. When the chip operates in normal light mode, we first set the D flip-flop to 0 via the RN signal. Subsequently, upon photon arrival, the pixel outputs a high-level signal. This signal is fed into the D flip-flop, causing its output to toggle, thereby achieving the function of TFF. The subsequent TBUF then latches the data, functioning identically to DFF.

**Figure 3:**
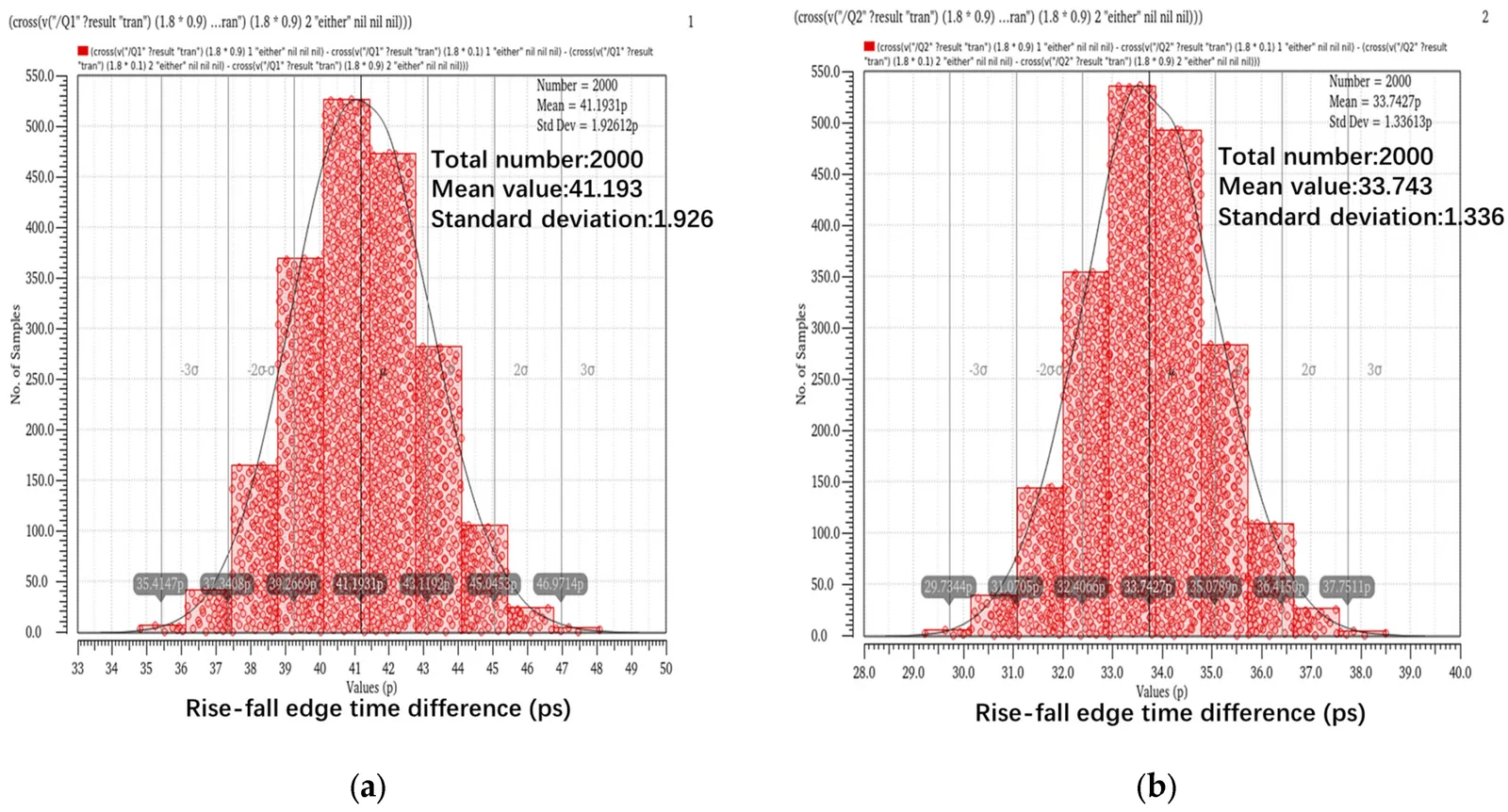
Monte Carlo simulation of the difference between the rise time and fall time at the output of the D flip-flop. The horizontal axis represents the rise–fall edge time difference (ps), and the vertical axis represents the number of sampling points (count). (**a**) Simulation results of the D flip-flop from the standard cell library; (**b**) simulation results of the optimized D flip-flop.

According to Equation (4), the difference between the rise time and fall time of the D flip-flop output affects the randomness of the output sequence. During the pixel design, we first performed a Monte Carlo simulation on the rise–fall time difference of the D flip-flop from the standard cell library. Two nodes of the D flip-flop output voltage signal were selected for calibration, corresponding to 10% and 90% of the high-level voltage, respectively. The rise time and fall time of the D flip-flop were measured based on the time interval between these two voltage nodes. The simulation results are shown in Figure 3a. Subsequently, we adjusted the aspect ratio of the MOSFETs in the D flip-flop to reduce the rise–fall time difference and performed another Monte Carlo simulation, with the results shown in Figure 3b. The simulation results indicate that the original D flip-flop exhibits a rise–fall time difference of 41.193 ps, which is reduced to 33.743 ps after adjustment. The quenching circuit we designed takes approximately 30 ns to complete the quenching process upon detecting a photon. The error introduced by the rise–fall time difference accounts for 33.743 ps/30 ns = 0.112%, which is sufficiently small to meet the requirements of array design.

### 3.2. Pixel Array Design

**Figure 4:**
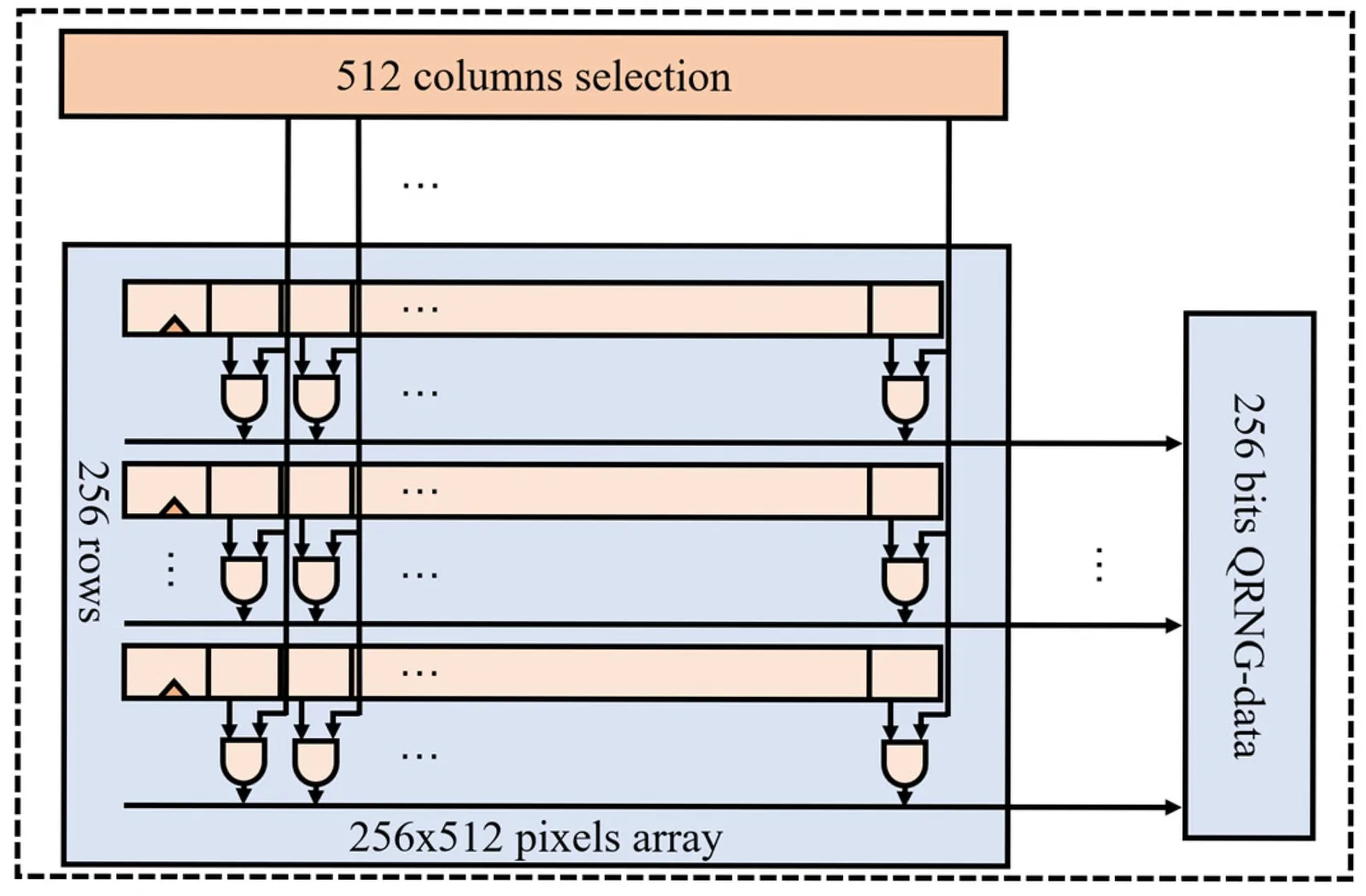
Schematic diagram of pixel array. The top section is the selection circuit, and the right is the channel control circuit.

The pixel structure of the chip is shown in Figure 4. Our designed chip comprises 256 × 512 pixels, arranged in 256 rows and 512 columns. The chip has 256 output channels and leverages a rolling-sampling mechanism to perform time-division multiplexing on the 512-column direction. Each row of pixels terminates with a TBUF acting as a control switch, enabling each pixel to generate a random bit. The rolling-sampling architecture temporally interleaves outputs from different pixels. The present work focuses on the temporal autocorrelation of the individual QRFF output and the statistical properties of the aggregated raw bitstream; a dedicated characterization of inter-pixel spatial correlation is not included in the present measurements. Based on the conclusion from Equation (10), we employ sequential inter-pixel readout to increase the temporal separation between successive observations of the same pixel. At each column-selection cycle, one pixel from each of the 256 rows is sampled simultaneously, generating 256 output bits. The column index advances sequentially from 0 to 511. Therefore, each pixel is sampled once every 512 column cycles, yielding an effective sampling interval of T_SAMP_ = 512T_COL_. This ensures each row of pixels continuously generates random bit sequences, with periodic sampling performed on each pixel. We prevent one single pixel outputting multiple consecutive bits. Instead, each pixel within a row’s bit sequence maintains a 512-bit interval between its own successive outputs.

**Figure 5:**
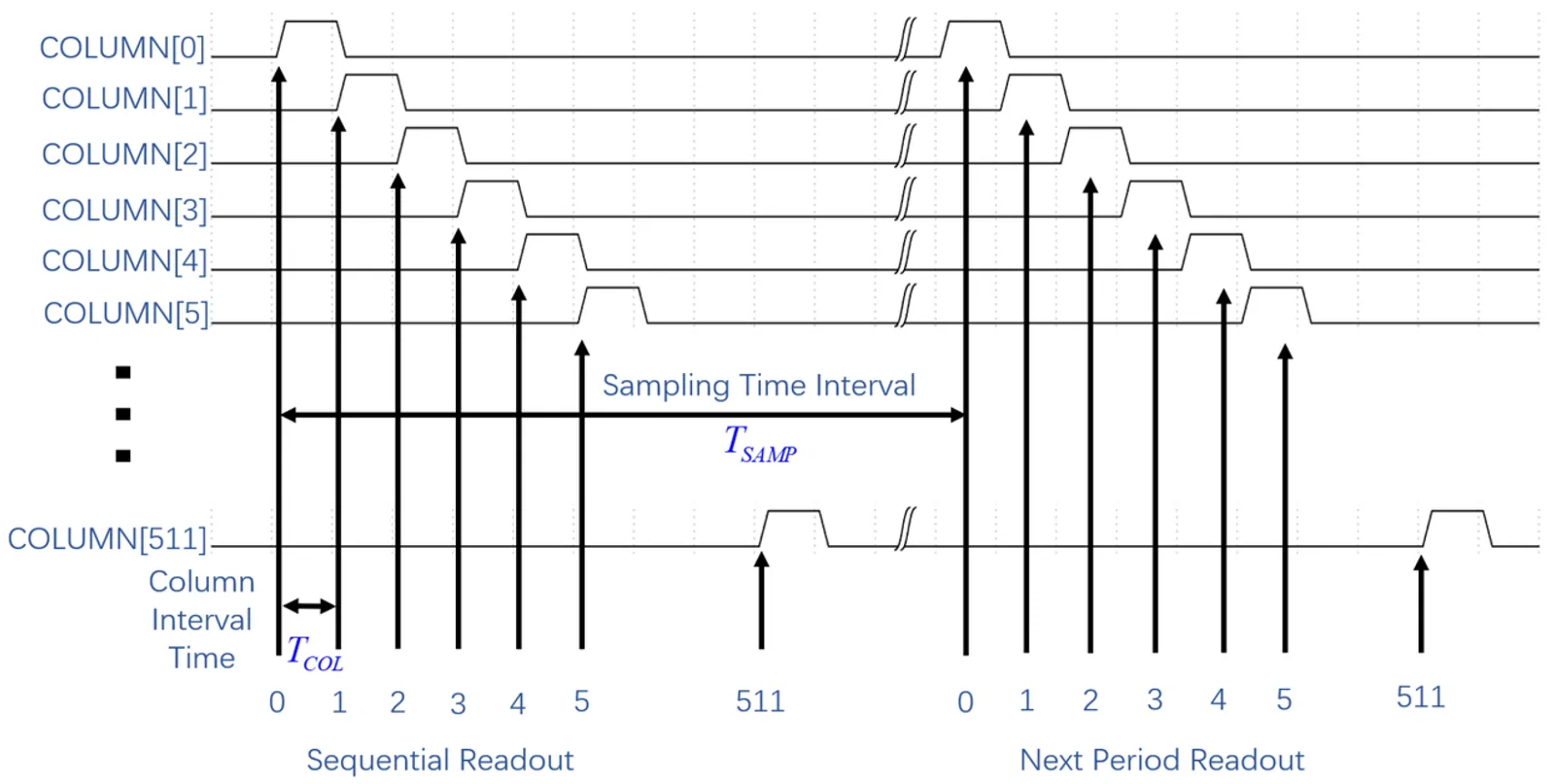
Pixel array readout waveform. The array begins with COLUMN [0], and after a column interval time ***T_COL_***, the next column is read. The interval between two reads of the same pixel is the sampling time interval ***T_SAMP_*** = ***T_COL_*** × 512.

The pixel readout architecture adopts a sequential scanning principle analogous to the rolling-shutter readout commonly used in CMOS image sensors [18]. In the proposed QRNG, this concept is adapted to sequential column selection across the SPAD array, as illustrated in Figure 5. The array begins with COLUMN [0], and after a column interval time ***T_COL_***, the next column is read. After reading COLUMN [511], the process resumes from COLUMN [0]. The interval between two consecutive reads of the same pixel is the sampling time interval ***T_SAMP_*** = ***T_COL_*** × 512. According to Equation (10), the sampling interval directly affects the temporal correlation between successive observations of the same pixel. In the column direction, we designed 256 rows of pixels, representing our chip’s 256 output channels capable of simultaneously generating 256 random bit sequences. The TBUF at the end of each row of pixels acts as a switch to control whether the random bit sequence generated by that row can be output. When TBUF is enabled, the pixel row it controls outputs the random bit sequence. When fewer channels are required in an application, TBUF can be disabled to reduce the number of output sequences.

Overall, the designed SPAD QRNG chip achieves high-speed, 256-channel parallel output while maintaining a simple pixel layout and stable operational functionality. By leveraging the rolling-sampling time-division multiplexing architecture, the proposed chip substantially increases the effective sampling interval of each pixel, thereby reducing temporal correlation by increasing the effective per-pixel sampling interval. This design enables the continuous generation of raw random bit sequences even under relaxed, low-illumination conditions. To evaluate the chip’s operational performance, validate the theoretical decorrelation model, and verify the statistical randomness of the generated sequences across various operating parameters, an experimental testing platform was constructed, as detailed in Section 4.

## 4. System Design and Testing Platform

### 4.1. System Design

**Figure 6:**
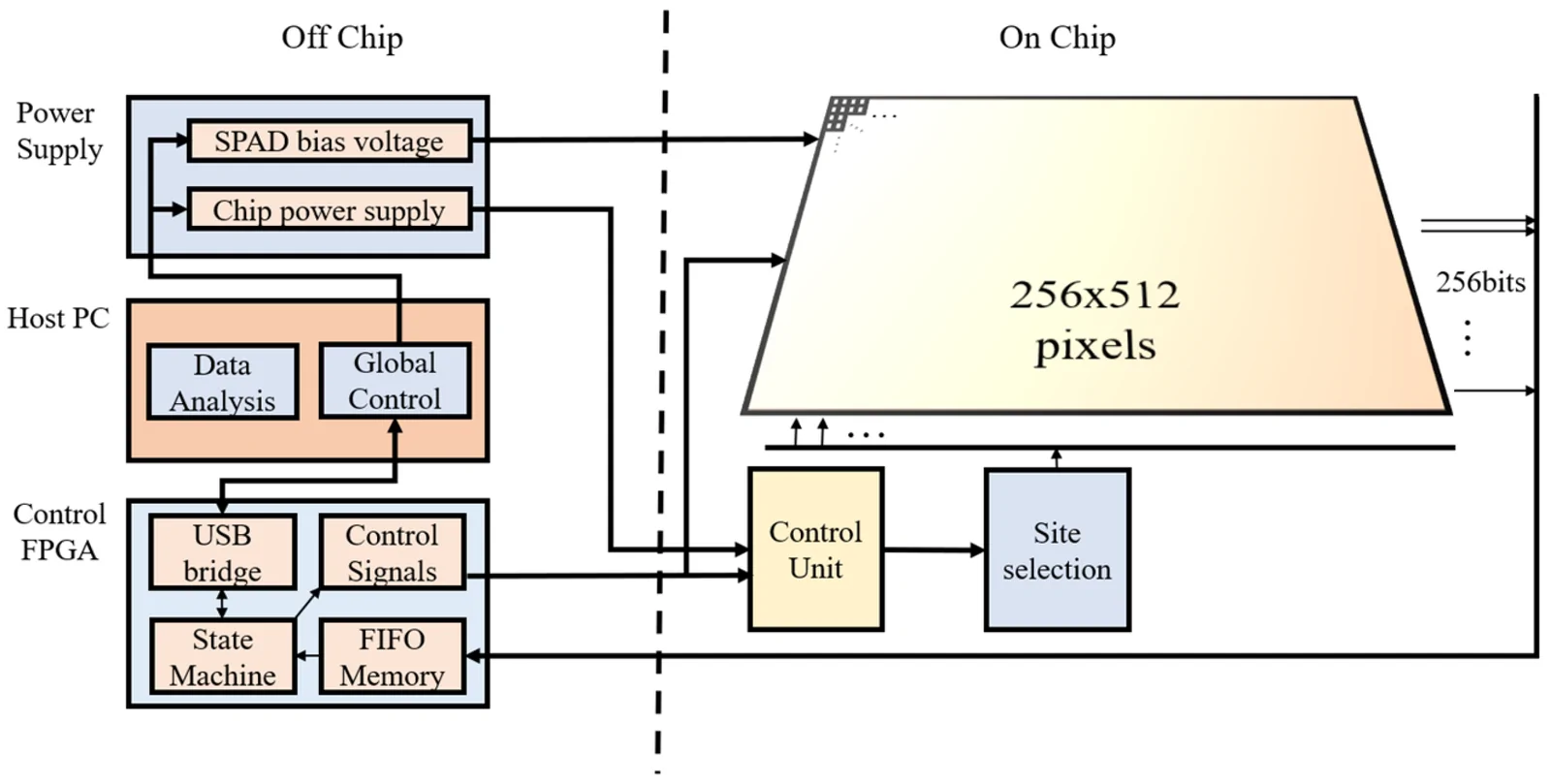
System design diagram. The left side shows the off-chip components, including the FPGA, host computer, power supply system, etc. The right side shows the on-chip components. The off-chip components are connected to the on-chip components via signal lines.

The system design of the random number chip is shown in Figure 6. The entire system is divided into on-chip and off-chip components. The on-chip portion consists of our designed random number generation chip, while the off-chip portion comprises the supporting system, primarily including the control FPGA, host computer, and power supply. The FIFO within the control FPGA temporarily stores the random bits generated by the chip. An internal state machine generates control signals, transmitting data to the host computer via the USB communication protocol. The power supply primarily provides the negative voltage required for the SPAD device’s operation and the working voltage needed for the logic modules. In this system, the random number generation chip functions normally, rapidly producing random bit sequences while simultaneously delivering them to the required devices.

### 4.2. Testing Platform

**Figure 7:**
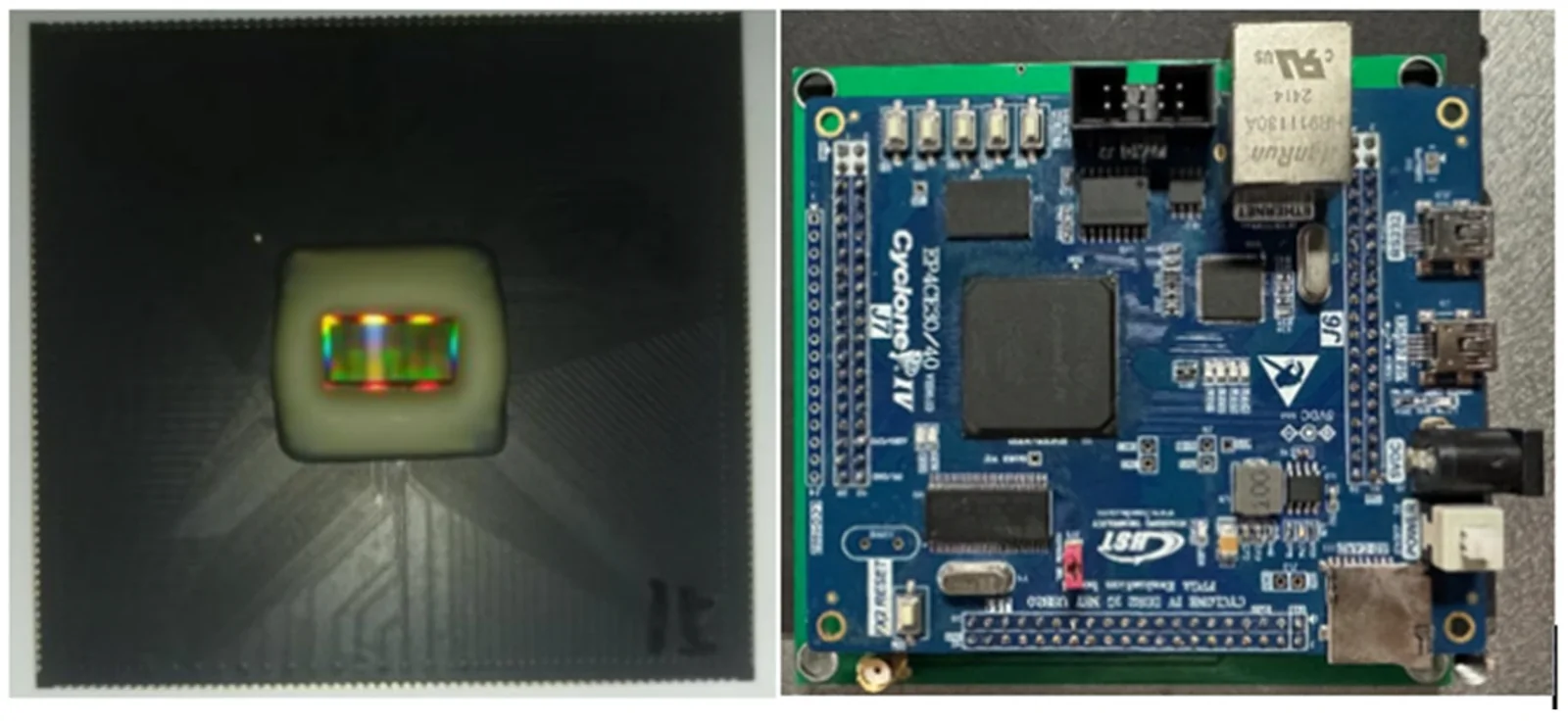
Chip and board system: on the left is our packaged chip, and on the right is the FPGA system used.

Figure 7 shows our chip after tape-out, containing 256 × 512 = 131,072 pixels. Its structure is as described earlier. On the right is our board-level system, comprising a custom-designed PCB and an FPGA. The chip is soldered to one side of the PCB, which is then inserted into the FPGA.

**Figure 8:**
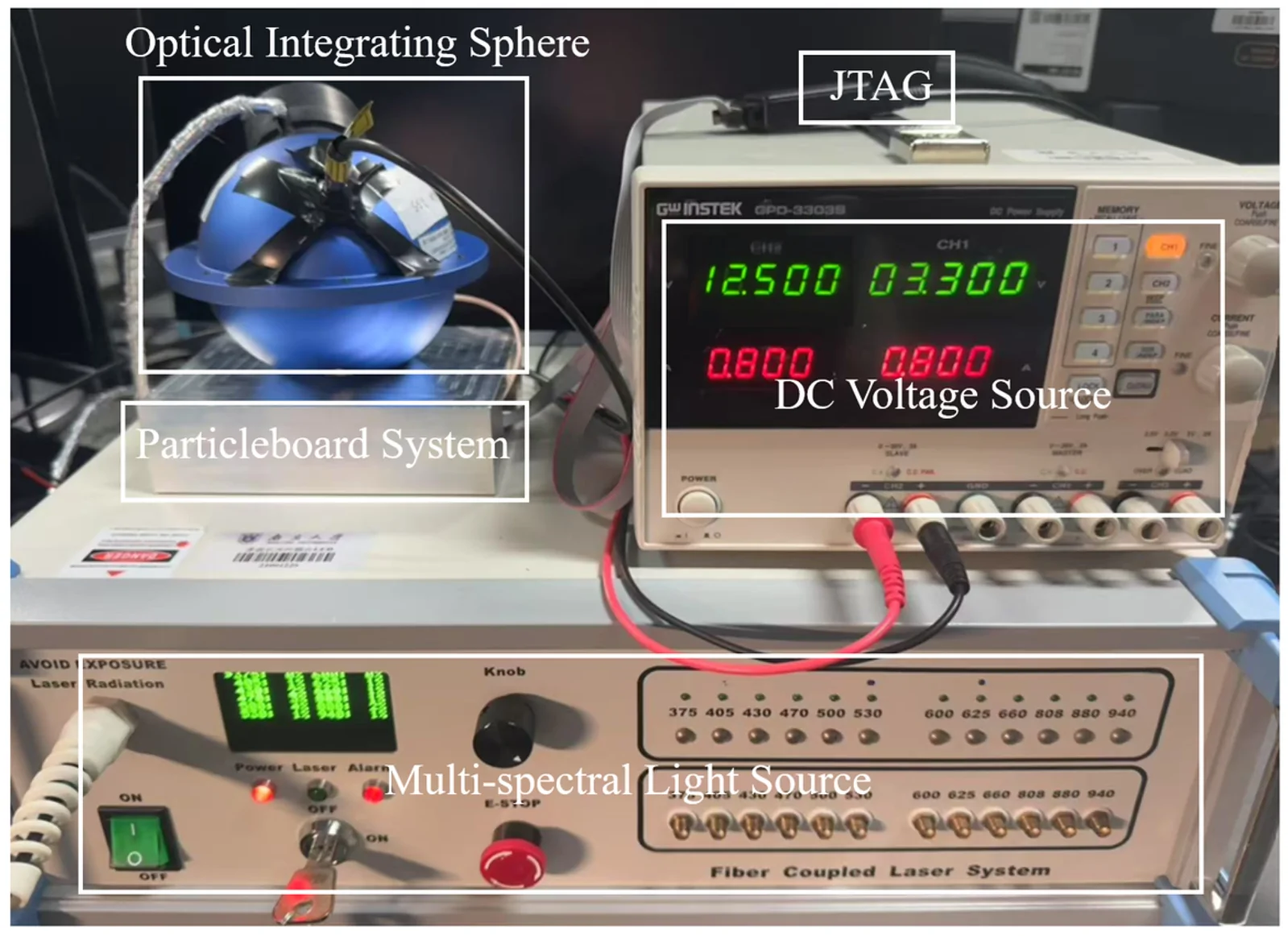
Integrated test system. The testing system consists of five components: optical integrating sphere, particleboard system, multi-spectral light source, DC voltage source and JTAG.

Figure 8 depicts our comprehensive testing system, primarily comprising the board-electrode system, an integrating sphere, a DC voltage source, a light source, and a host computer. An optical integrating sphere is mounted atop the metal enclosure and connected to the light source, ensuring uniform illumination across the chip. The plate-anode system’s power requirements are supplied by the power source. It connects to the host computer via JTAG and USB cables for generating and transmitting random bit sequences.

## 5. Chip Testing and Results Analysis

We conducted comprehensive experiments to evaluate the random number generation performance of the fabricated chip and investigate how key operating parameters impact sequence entropy and temporal correlation. First, we verified the statistical randomness of the raw bit streams generated under standard indoor ambient illumination using the NIST SP 800–22 test suite. Next, under constant illumination, we systematically swept the pixel sampling interval to measure the single-pixel serial autocorrelation coefficient (a_1_), thereby experimentally characterizing the temporal-decorrelation behavior associated with the rolling-sampling architecture. Subsequently, utilizing an integrating sphere, multi-wavelength optical source, and power meter, we varied the incident optical power at 530 nm to characterize the temporal correlation under reduced illumination and identify the lowest tested optical-power point satisfying the adopted correlation reference. Below, we present the detailed experimental results and data analysis. Additional experimental details and supporting results are provided in the Supplementary Materials.

### 5.1. Randomness Detection of Output Sequences Under Normal Conditions

We conducted NIST tests on the random numbers generated by the chip to statistically characterize the raw bitstreams. Each NIST sequence contains 10^6^ bits, and the significance level is a = 0.01. The test results are shown in Table 1. The system’s normal mode refers to light conditions with standard illumination (light power approximately 8 W/m^2^), the excess bias voltage of 2 V. We utilized an FPGA to control the operating state of the chip, completing a sampling operation every 80 ns to simultaneously generate 256 random bits. With 256 parallel channels, the random number generation rate reaches 3.2 Gb/s. Under these conditions, the column scanning period T_COL_ is 80 ns and the single-pixel sampling interval T_SAMP_ is 40.96 μs. Under these operating conditions, the generated raw bitstreams pass all 15 NIST SP 800–22 tests at the specified significance level without digital post-processing. It should be noted that the reported 3.2 Gb/s rate represents the aggregate raw hardware output bitrate rather than an independently established entropy rate of the full array. The NIST SP 800–22 results and serial-correlation measurements provide statistical characterization of the raw output but do not constitute a formal min-entropy or entropy-rate assessment.

### 5.2. Effects of Pixel Sampling Interval on Serial Autocorrelation

To experimentally evaluate the temporal decorrelation behavior predicted by the approximate model in Section 2.2, we measured the one-bit-lag serial autocorrelation coefficient a_1_ for different sampling intervals T_SAMP_. By controlling the FPGA readout timing, we swept T_SAMP_ across a wide range while maintaining constant ambient illumination. For each pixel, a long sequence of raw output bits X = {x_1_, x_2_, ..., x_N_} was captured, and the serial autocorrelation coefficient a_1_ was calculated as [19]

(11) $$\;a_{1}=\frac{\sum_{j=1}^{N-1}\left(x_{j}-x_{bar})(x_{j+1}-x_{bar}\right)}{\sum_{j=1}^{N}{\left(x_{j}-x_{bar}\right)}^{2}}$$

where x_bar_ is the sample mean of the generated sequence.

**Figure 9:**
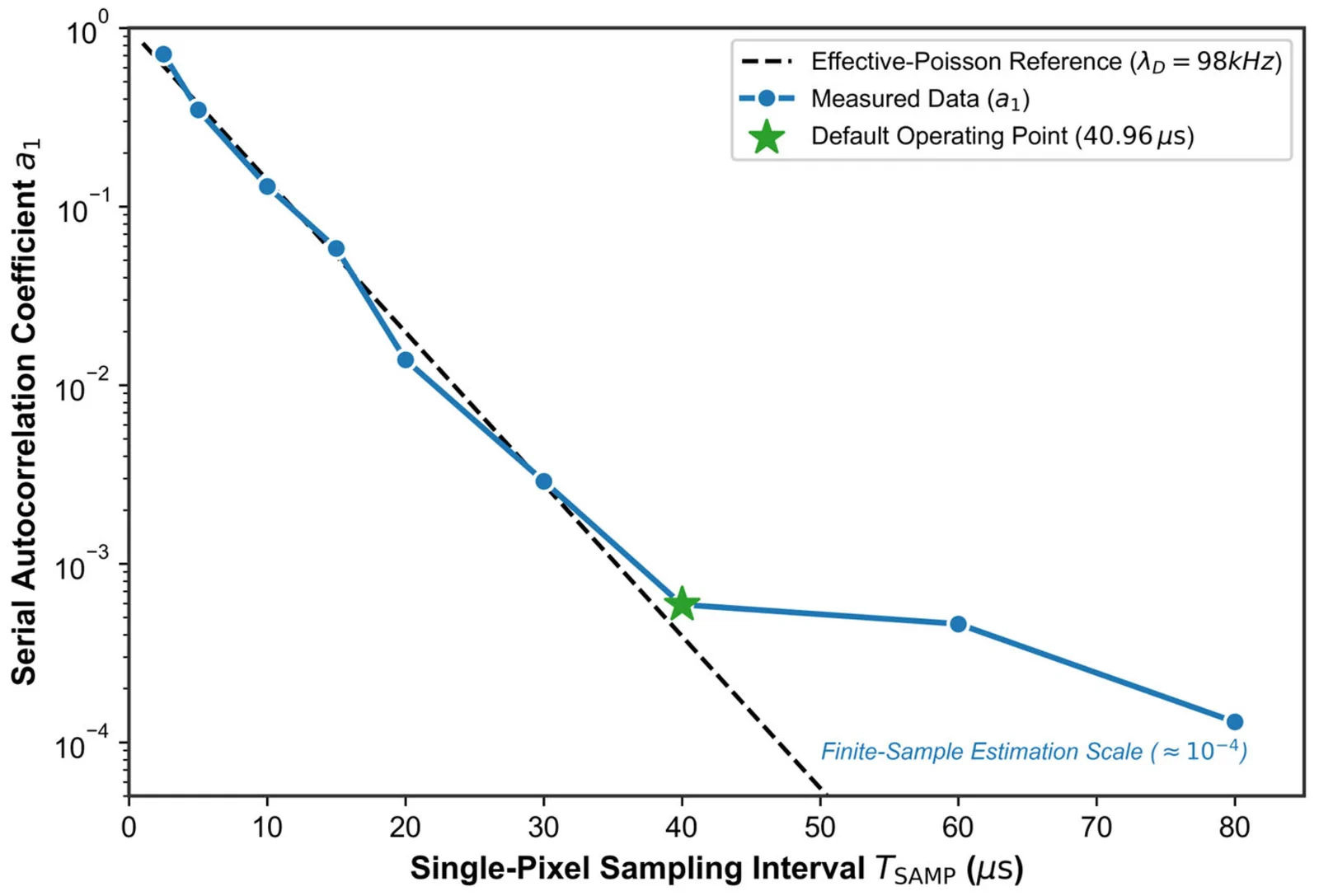
Measured one-bit-lag serial autocorrelation coefficient a_1_ as a function of the single-pixel sampling interval T_SAMP_ under a fixed illumination condition. The dashed curve denotes the effective-Poisson (EP) reference a_1_^EP^, calculated using the measured photon detection rate of λ_D_ = 98 kHz.

In the measurements, N = 10^8^ raw bits were collected from a single SPAD pixel for each sampling interval. The measured photon detection rate of the selected pixel was approximately λ_D_ = 98 kHz. As shown in Figure 9, a_1_ decreases rapidly with increasing T_SAMP_, demonstrating the effectiveness of extending the sampling interval in reducing the temporal correlation of the generated bit sequence.

The reduction in a_1_ with increasing T_SAMP_ should not be attributed exclusively to the suppression of SPAD dead-time memory. Two effects occur simultaneously as T_SAMP_ increases. First, the sampling interval becomes longer relative to the characteristic detector-memory timescale, thereby reducing the relative influence of short-time detector memory. Second, a longer sampling interval accumulates more photon-detection events between successive samples, which changes the even–odd parity statistics and can reduce the TFF correlation even for an ideal Poisson detector. These two effects are coupled through T_SAMP_ and cannot be independently quantified from the present fixed-illumination dataset. Therefore, the measured decrease in a_1_ demonstrates the combined effectiveness of increasing the sampling interval for temporal decorrelation.

At short sampling intervals, the measured a_1_ values exhibit a noticeable deviation from the EP reference. This behavior is consistent with the limitations of the effective-Poisson approximation, which does not fully capture the non-Poisson renewal statistics and residual detector memory at short observation intervals. As T_SAMP_ increases, the measured serial autocorrelation decreases from 0.71449 at 2.5 μs to 0.01387 at 20 μs, and further to 0.00059 at 40 μs. The 40 μs value is an experimentally measured sampling interval and is close to the nominal operating sampling interval of 40.96 μs used in the implemented rolling-sampling architecture, which is determined by 512 × 80 ns.

For T_SAMP_ > 40 μs, the measured a_1_ remains on the order of 10^−4^, whereas the EP reference continues to decrease toward zero. This divergence is not interpreted as a fundamental physical correlation floor. Since each measurement contains N = 10^8^ bits, the characteristic finite-sample estimation scale is

(12) $$\;\frac{1}{\sqrt{N}}\approx 10^{-4}$$

When the true correlation becomes sufficiently small, the measured autocorrelation coefficient is therefore increasingly affected by finite-sequence statistical uncertainty, together with residual detector and non-Poisson effects that are not captured by the EP approximation. The observed 10^−4^-level behavior is thus regarded as an empirical estimation scale for the present finite-length measurement rather than as a fundamental lower bound of the generated random sequence.

Overall, the experimental results demonstrate that increasing T_SAMP_ substantially reduces the measured temporal autocorrelation. The results are consistent with the combined effects of reduced relative detector-memory influence and increased photon-count accumulation discussed in Section 2.2. Although the present fixed-illumination experiment does not independently separate these two contributions, it experimentally verifies that the rolling-sampling architecture can substantially suppress temporal correlation without digital post-processing.

### 5.3. Low-Optical-Power Characterization at 530 nm

To evaluate the operation of the proposed QRNG under reduced optical illumination, the incident optical power was varied at a fixed wavelength of 530 nm. This wavelength was selected because it corresponds to the peak photon detection efficiency (PDE = 48.17% at an excess bias of 2 V) of the SPAD used in this work. For each optical-power level, N = 10^8^ raw bits were acquired from the same single SPAD pixel used for the sampling-interval characterization in Section 5.2, and the lag-1 serial autocorrelation coefficient a_1_ was calculated according to Equation (11). During the measurement, the rolling-sampling architecture was operated under the same nominal timing condition, with T_COL_ = 80 ns and T_SAMP_ = 40.96 μs. The lag-1 serial autocorrelation coefficient a_1_ of the raw output sequence was evaluated at incident optical powers of 5.6, 8.4, 11.6, and 14.9 μW. The incident optical power reported in this section is determined from the irradiance measured inside the integrating sphere. Specifically, the measured optical irradiance (power per unit area) is multiplied by the photosensitive area of the SPAD array to obtain the total optical power incident on the detector area.

**Figure 10:**
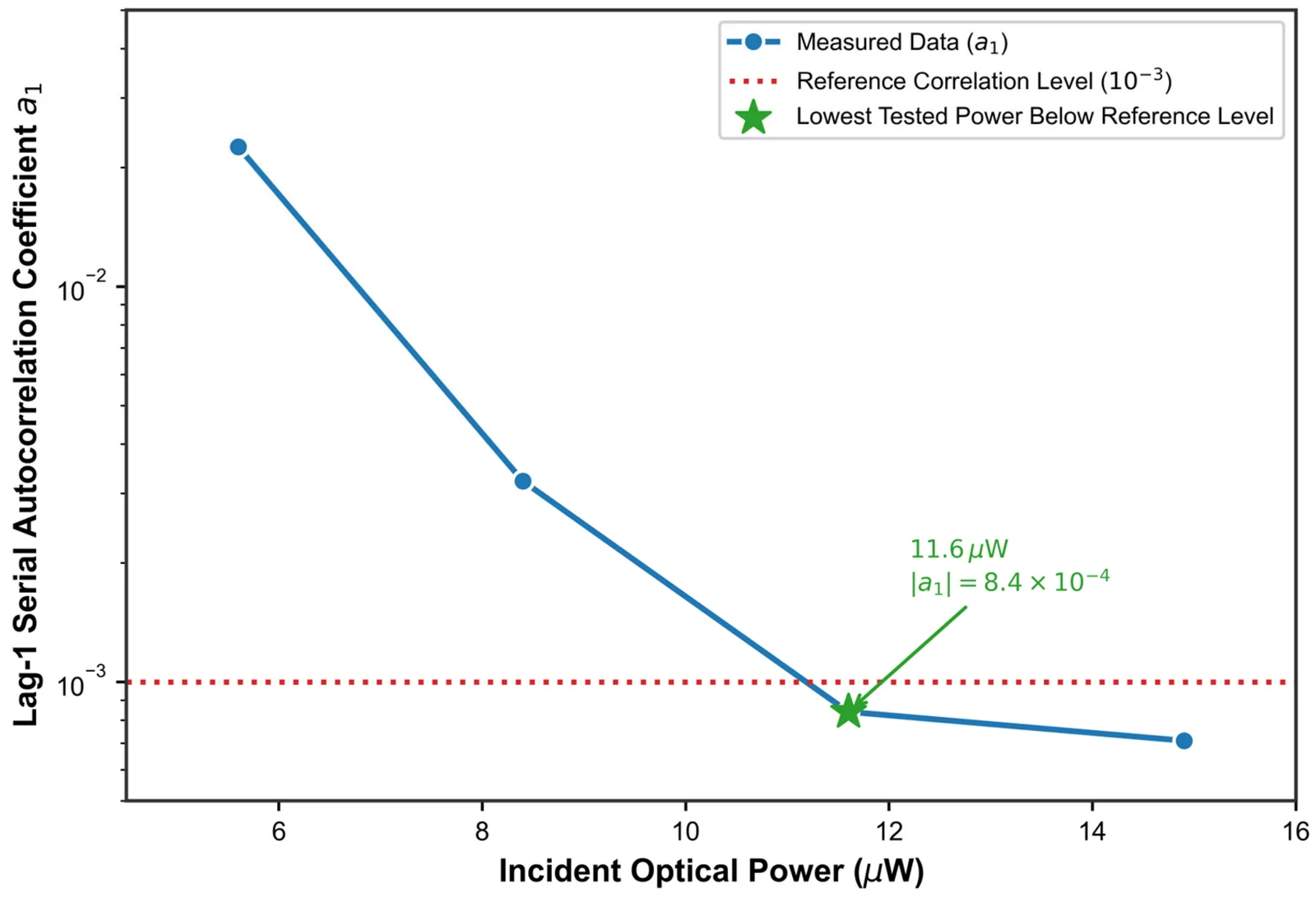
Measured magnitude of the lag-1 serial autocorrelation coefficient (a_1_) as a function of incident optical power at 530 nm. The dotted line denotes the reference correlation level of 10^−3^ adopted for comparison with the prior QRFF characterization in [9].

Figure 10 shows the measured magnitude of the lag-1 serial autocorrelation coefficient as a function of incident optical power. At 5.6 μW, the measured correlation is a_1_ = 0.02256. Increasing the optical power to 8.4 μW reduces a_1_ to 0.00322. A further increase to 11.6 μW reduces the measured correlation to 0.00084, while at 14.9 μW, a_1_ further decreases slightly to 0.00071. These measurements demonstrate a pronounced reduction in temporal correlation as the incident optical power is increased.

This behavior can be understood from the photon-event statistics of the QRFF. At lower optical power, the photon-detection rate is reduced, resulting in fewer random TFF transitions between two successive samples of the same pixel. Consequently, the sampled TFF state retains a stronger temporal dependence. Increasing the optical power increases the number of photon-detection events accumulated within each sampling interval and thereby modifies the even–odd parity statistics of the TFF transitions, reducing the measured lag-1 correlation. This observation is consistent with the finite-event-rate dependence discussed in Section 2.1 and Section 2.2. As discussed previously, the measured decorrelation should not be attributed exclusively to the suppression of SPAD dead-time effects.

For comparison with the closely related QRFF implementation reported in [9], Figure 10 includes a_1_ = 10^−3^ as a reference correlation level. In [9], a correlation magnitude of 10^−3^ was adopted as a performance benchmark and was associated with an estimated min-entropy of approximately 0.9986 bit/bit with the correlation-based entropy model used in that work. The 10^−3^ level is therefore used here as a reference benchmark for comparison with prior QRFF characterization rather than as a universal cryptographic-security threshold.

Among the four investigated illumination levels, 11.6 μW is the lowest tested incident optical power for which the measured lag-1 autocorrelation falls below this reference level, with a_1_ = 0.00084. The corresponding Shannon information entropy of the measured raw sequence is 0.9999984 bit/bit. At 14.9 μW, the correlation decreases only slightly further to 0.00071, indicating that the principal improvement within the investigated optical-power range occurs between 5.6 and 11.6 μW. Therefore, 11.6 μW is identified as the lowest demonstrated optical-power operating point satisfying the adopted correlation reference under the present measurement conditions.

## 6. Conclusions

In this work, we designed and fabricated a high-speed, multi-channel SPAD-based quantum random number generator in a standard CMOS process. The chip integrates a 256 × 512 SPAD-QRFF array with 256 parallel output channels and employs a rolling-sampling time-division multiplexing architecture across 512 columns. This architecture extends the per-pixel sampling interval to 40.96 μs while maintaining an aggregate raw output bitrate of 3.2 Gb/s without digital post-processing.

Experimental characterization demonstrates that increasing the per-pixel sampling interval substantially reduces the measured temporal autocorrelation of the raw output. The observed decorrelation is attributed to the combined effects of increased photon-event accumulation between successive samples and the reduced relative influence of short-time detector memory rather than exclusively to SPAD dead-time suppression. Under standard indoor illumination of approximately 8 W/m^2^, the 3.2 Gb/s raw bitstream passes all 15 NIST SP 800–22 statistical tests at the specified significance level.

The low-optical-power behavior was further characterized under controlled 530 nm illumination. The measured lag-1 serial autocorrelation coefficient decreases from 0.02256 at 5.6 μW to 0.00084 at 11.6 μW. Among the investigated illumination levels, 11.6 μW is the lowest tested incident optical power for which a_1_ falls below the 10^−3^ reference correlation level adopted for comparison with prior QRFF characterization. This result demonstrates low-optical-power operation of the fabricated chip under the specified measurement conditions.

As summarized in Table 2, the proposed architecture adopts a different throughput-scaling strategy from prior integrated SPAD QRNGs. Rather than maximizing the generation rate of each individual pixel, the proposed design uses large-scale spatial parallelism to increase the temporal separation between successive observations of the same pixel while maintaining multi-gigabit aggregate throughput. This represents an architectural trade-off between spatial resources, per-pixel sampling rate, and aggregate generation rate. The present results demonstrate that rolling sampling provides a practical hardware approach for combining long per-pixel revisit intervals with high aggregate raw-bit throughput in integrated SPAD-based QRNGs.

## Figures and Tables

**Table 1:** NIST SP 800–22 statistical randomness test results under standard indoor illumination.

Test	*p*-Value	Pass Rate	Result
Frequency	0.6803	99%	Passed
Block Frequency	0.7887	100%	Passed
Cumulative Sums	0.2811	98%	Passed
Runs	0.2672	98%	Passed
Longest Run	0.5012	99%	Passed
Rank	0.6758	100%	Passed
FFT	0.7048	100%	Passed
Non Overlapping Template	0.3799	99%	Passed
Overlapping Template	0.6512	98%	Passed
Universal	0.8767	99%	Passed
Approximate Entropy	0.5238	98%	Passed
Random Excursions	0.1168	97%	Passed
Random Excursions Variant	0.2625	98%	Passed
Serial	0.4199	99%	Passed
Linear Complexity	0.2153	99%	Passed

**Table 2:** Comparison with published integrated SPAD-based random number generators.

Ref. and Year	Circuit Implementation	Array Size	Bitrate	Bitrate per Pixel	Post-Processing
This work	QRFF + rolling sampling	256 × 512	3.2 Gb/s	24.4 Kb/s	None
[9] Keshavarzian. 2023	QRFF	40 × 70	3.3 Gb/s	1.18 Mb/s	None
[20] Regazzoni, 2021	Vector matrix multiplication	128 × 128	400 Mb/s	0.024 Mb/s	None
[21] Acerbi, 2018	Frame readout	1	0.1 Mb/s	0.1 Mb/s	None
[22] Xu, 2018	Inter-arrival arbiter	16 × 16	18 Mb/s	0.07 Mb/s	None
[23] Burri, 2013	Frame readout	512 × 128 × 2	5 Gb/s	0.04 Mb/s	VN filter

## Data Availability

Data is contained within the article or Supplementary Material.

## Supplementary Materials

The following supporting information can be downloaded at: https://www.mdpi.com/article/10.3390/s26196350/s1, Supplementary File S1.
